# The conundrum of the giant condyloma: is it carcinoma?

**DOI:** 10.1093/jscr/rjae668

**Published:** 2024-10-22

**Authors:** Hyung C Kim, James D Schlenker, Amber B Post, Nicholas S Agoff, Timothy Feldmann, Vlad V Simianu

**Affiliations:** Department of General Surgery, Virginia Mason Franciscan Health, 1100 9th Ave, Seattle, WA 98101, United States; Department of Plastic Surgery, Virginia Mason Franciscan Health, 1100 9th Ave, Seattle, WA, United States; Department of Radiation Oncology, Virginia Mason Franciscan Health, 1100 9th Ave, Seattle, WA, United States; Department of Pathology, Virginia Mason Franciscan Health, 1100 9th Ave, Seattle, WA, United States; Department of General Surgery, Multicare Capital Medical Center, 3920 Capital Mall Drive SW, Olympia, WA, United States; Department of General Surgery, Virginia Mason Franciscan Health, 1100 9th Ave, Seattle, WA 98101, United States; Section of Colorectal Surgery, Virginia Mason Franciscan Health, 1100 9th Ave, Seattle, WA, United States

**Keywords:** verrucous carcinoma, colorectal surgery, plastic and reconstructive surgery, radiation oncology

## Abstract

Perineal verrucous carcinoma is a rare variant of squamous cell carcinoma that is mainly treated with surgical excision. In this case report, we present a 58-year-old man with human immunodeficiency virus who presented with an extraordinarily large perineal mass that was ultimately found to be verrucous carcinoma in association with giant condyloma acuminata. He was treated with a wide local excision followed by staged abdominoperineal resection and fasciocutaneous flap reconstruction. In the post-operative course, the patient developed relatively short interval recurrence which was successfully managed with salvage radiotherapy. He is now post-radiation without evidence of recurrence.

## Introduction

Perineal verrucous carcinoma (VC) is a rare form of squamous cell carcinoma (SCC) that presents with cauliflower-like growth on anogenital regions such as penis, vulva, vagina, or perianal regions [1]. The main distinction from giant condyloma acuminanta (GCA), a similar lesion, is that VC is not associated with human papilloma virus infection (HPV) [2]. We present a rare case of VC arising in association with GCA, multidisciplinary approach to treatment, and long-term outcome.

## Case report

**Figure 1:**
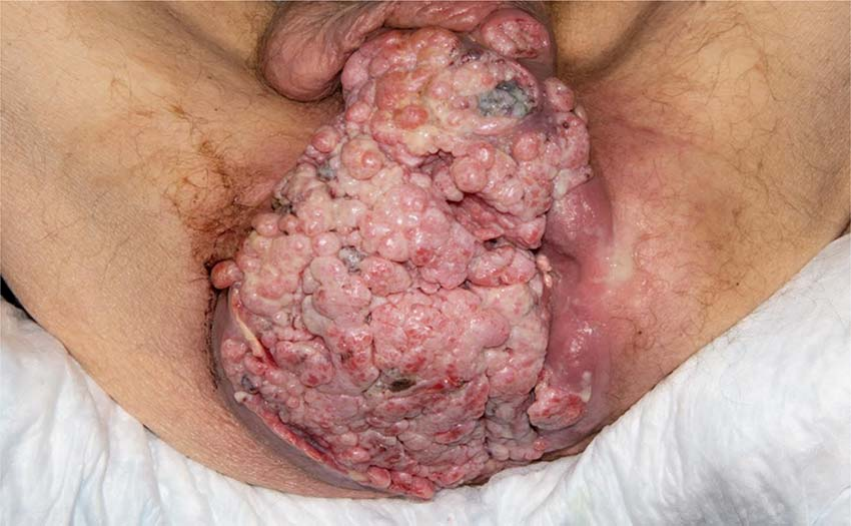
Perineal mass as examined in the lithotomy position.

A 58-year-old man with history of human immunodeficiency virus (HIV) on Genvoya and type 2 diabetes presented with a 17-month history of progressively enlarging fungating anal mass. He underwent multiple biopsies of the mass which showed only anal intraepithelial neoplasia I (AIN I), consistent with condyloma. However, given the extent of the mass (Fig. 1), incontinence, ongoing bleeding, and sepsis (erythema and purulent drainage), malignancy needed to be ruled out and definitive treatment was necessary.

**Figure 2:**
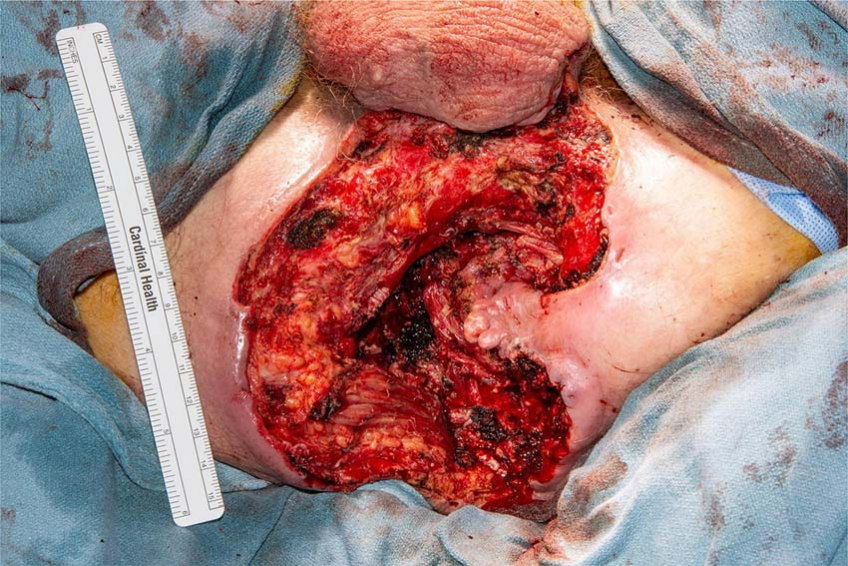
Perineal wound after wide local excision, patient in the lithotomy position.

The patient was given blood transfusions, started on broad spectrum antibiotics, and urgently brought to the operating room to undergo exam under anesthesia which showed purulent drainage from the mass, but it was found to be limited to the right side of anal canal without intra-anal condyloma, and circumferentially clear of the dentate line. Biopsies taken at that time again showed AIN I. Staging workup for occult anal cancer by CT showed no evidence of metastatic disease or inguinal adenopathy. Latest CD4 count was 667 cell/mcL. After review at multidisciplinary cancer conference, the recommendation was to perform wide local excision to obtain definitive diagnosis as well as source control for ongoing infection (Fig. 2). Wound was temporized with wound vacuum-assisted closure (VAC) placement.

**Figure 3:**
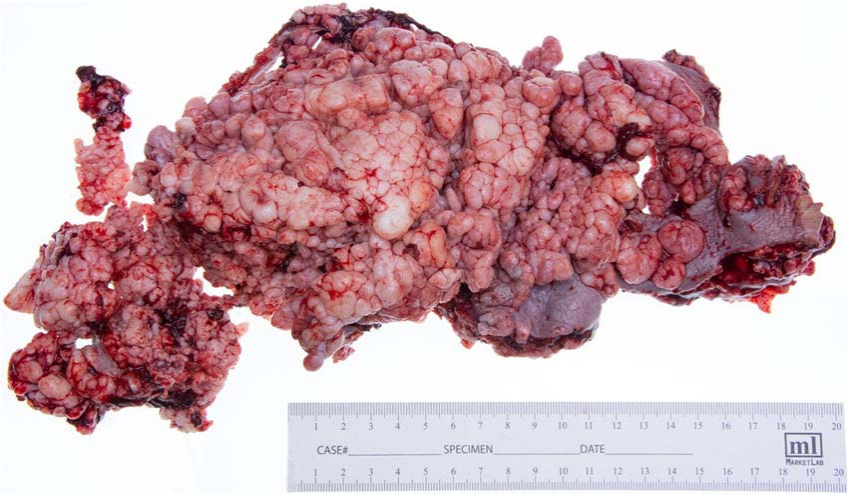
Gross photo of wide local excision, measuring 28 × 13 × 6 cm.

**Figure 4:**
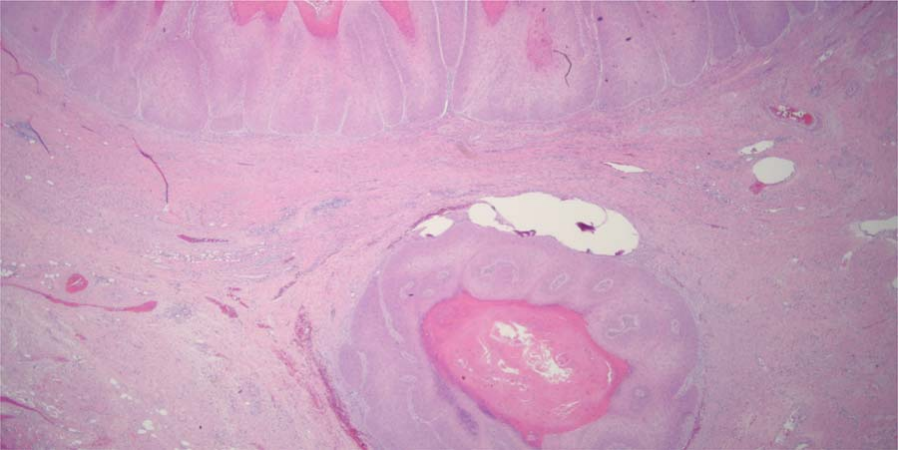
Histologically the specimen was a well-differentiated squamous cell carcinoma with pushing borders without any infiltrative invasion, specifically distinguishing it from conventional invasive squamous cell carcinoma.

The patient underwent serial debridements and wound VAC changes until final pathology was confirmed as VC arising in association with GCA measuring 28 × 13 × 6 cm (Fig. 3) without any lymphatic or vascular invasion. The tumor had pushing borders without any invasion, which distinguishes it from conventional invasive SCC (Fig. 4).

**Figure 5:**
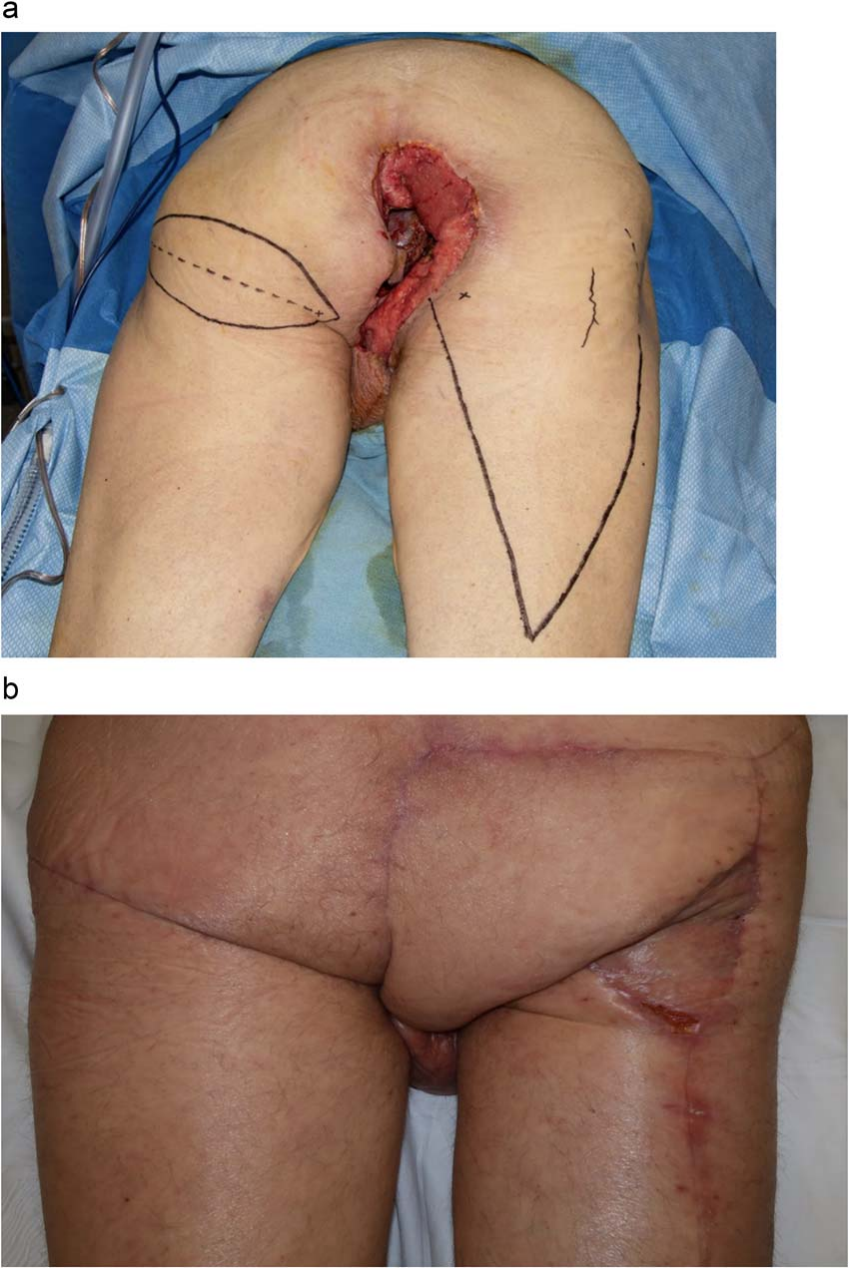
With patient in prone position, (a) marked pedicle flap reconstructions. Using an inferior gluteal fold fasciocutaneous flap on the left side, and gluteal thigh flap on the right side. (b) Final reconstruction result at 2 weeks.

Considerations were given to the patient’s pre-existing fecal incontinence, large wound requiring complex reconstruction, and local lymph node evaluation. Multidisciplinary decision was made to proceed with robotic-assisted abdominoperineal resection (APR) followed by staged reconstruction. To fill the internal pelvic defect, pedicled left inferior gluteal fold fasciocutaneous flap was passed through the levators. For the sizable external defect, pedicled right gluteal thigh fasciocutaneous flap was used to cover the midline with extension to the scrotum (Fig. 5a and b). The patient was discharged 2 weeks after reconstruction.

**Figure 6:**
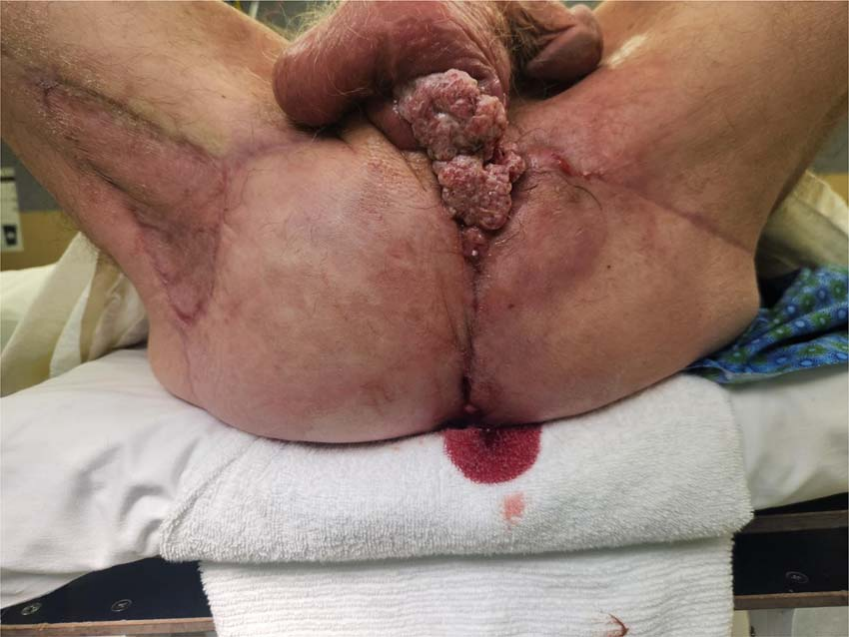
In lithotomy position, recurrence at the anterior/scrotal aspect of perineal incision, with recurrence identified also at posterior and right lateral aspect underneath flaps.

Unfortunately, at 3 months the patient re-presented with concern of recurrence. Restaging CT and PET scan still demonstrated no metastatic disease. CD4 count was now 160 cell/mcL. Patient underwent additional wide local excision, where the recurrence was found to be tracking underneath the flaps (Fig. 6).

After multidisciplinary review, the decision was made to pivot away from additional surgery to radiation, and patient underwent a total of 15 fractions of daily radiotherapy to a total dose of 4500 cGy to the perineum and post-surgical field. He completed treatment prior to discharge without complication.

**Figure 7:**
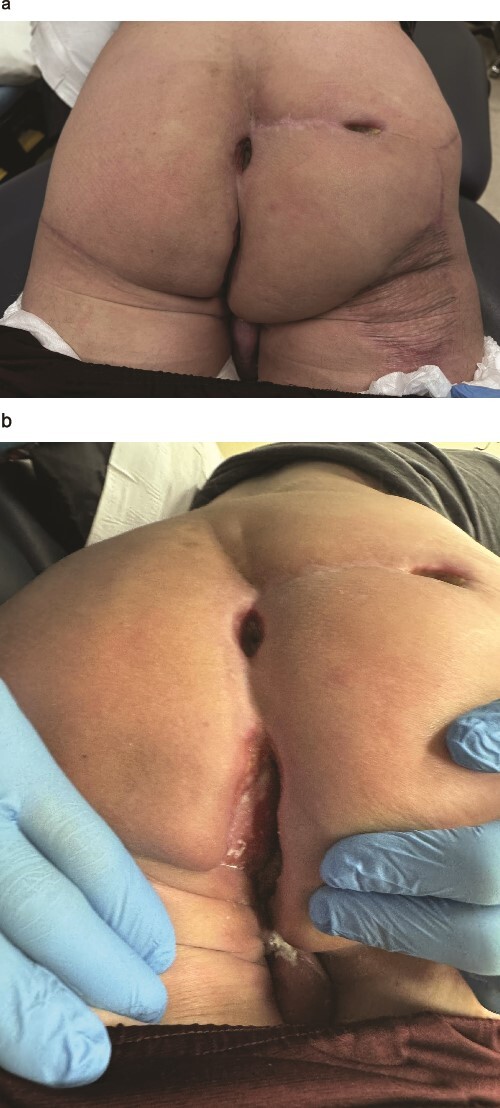
Wounds to perineal defect healing by secondary intention 6 weeks after radiation therapy. (a) Patient in prone position. (b) Patient in left lateral decubitus position

When seen in 6-week follow up after his latest admission, there was no evidence of recurrence and patient’s wounds have healed well (Fig. 7a and 7b).

## Discussion

The differential diagnosis for perineal condyloma includes GCA, VC, or invasive SCC. While GCA is associated with low-risk HPV (types 6 and 11), VC is not [2]. Histologically, VC has deep pushing margins without any invasion [3], though it can be locally aggressive, as seen in this case. Differentiating VC from conventional SCC is crucial for management and prognosis, but distinguishing between them can be subtle and difficult.

If the mass had been invasive SCC, treatment would have begun with chemoradiation first, with possible salvage APR for persistence or recurrence [4]. Resistance to chemoradiotherapy is linked to size of tumor and HIV [5, 6]. In contrast, primary treatment for VC is surgical excision [3]. Alternatively, neoadjuvant chemo-radiation therapy has been used to reduce the size of the mass to avoid colostomy and APR [7] but in this case, early excision was performed for diagnosis and sepsis control. APR was performed given that the patient had pre-existing fecal incontinence and to facilitate complex reconstruction in the irregular defect. While an APR would also be an oncologic operation for SCC, performance of a total mesorectal excision would not be expected to improve survival in VC.

Following APR, various local flap reconstruction options are available including rectus abdominis flaps, gracilis flaps, and fasciocutaneous flaps from gluteal and thigh regions. In this case, inferior gluteal fold fasciocutaneous flap based on the internal pudendal artery was used to fill the pelvis [8]. These flaps are versatile for vaginal reconstruction, coverage of pressure sores, and interposition for repair of perineal fistulae. They are ideal for perineal procedures that do not require an open abdominal approach because they are elevated using the same exposure from below. Bilateral flaps can be used for more extensive coverage. Here, right inferior gluteal fold flap was not available because the flap blood supply was compromised by the necessary resection. Right-sided gluteal thigh flap based on the descending branch of the inferior gluteal artery for resurfacing of the perineal defect was chosen to allow transfer of a large, thin, pliable fasciocutaneous flap from the posterior thigh to the perineum. The gluteal thigh flap remains sensate through the posterior femoral cutaneous nerve, aiding in protective pressure offloading while sitting.

Once the VC recurred and tracked inferior to the flaps, the multidisciplinary team and patient agreed to attempt non-surgical management. Given that salvage radiation has been previously described for rapidly growing recurrent perineal VC [9], the decision was made for the patient to undergo radiation therapy. Hypofractionated course of 4500 cGy radiation therapy in 15 fractions was chosen versus longer traditional fractionation due to patient’s social situation. Although hypofractionation can increase risk of fecal incontinence, this was not a concern for this patient.

This report highlights a challenging case given the size of the mass and the subsequent wound, concern for underlying invasive cancer that could not be definitively demonstrated on biopsy, and the multidisciplinary involvement for diagnosis, treatment, complex reconstruction, and salvage.
